# Implications of the pandemic for the construction of nurses’ identity based on the journalistic media

**DOI:** 10.1590/0034-7167-2022-0245

**Published:** 2023-04-07

**Authors:** Loiza Broering, Maria Itayra Padilha, Roberta da Costa, Maiara Suelen Mazera

**Affiliations:** I Universidade Federal de Santa Catarina. Florianópolis, Santa Catarina, Brazil

**Keywords:** Pandemic, COVID-19, Nursing, Professional Practice, Mass Media., Pandemia, COVID-19, Enfermería, Práctica Profesional, Medios de Comunicación de Masas., Pandemia, COVID-19, Enfermagem, Prática Profissional, Meios de Comunicação de Massa

## Abstract

**Objectives:**

to analyze the work of nurses portrayed in the journalistic media and its impact on the construction of professional nursing identity.

**Methods:**

this is qualitative, retrospective, descriptive and documentary research, with 51 reports from Folha de São Paulo. Time frame from March to December 2020. Thematic Content Analysis carried out from Claude Dubar’s theoretical perspective. Organization and coding of data performed with the help of ATLAS.ti^®^.

**Results:**

three categories emerged: Working conditions in the pandemic - a problem that worsened; Impacts of the pandemic on daily work; Feelings generated by the pandemic.

**Conclusions:**

despite adversities, such as the precariousness of health institutions, inadequate working conditions for nurses, lack of basic items of individual protection, negative feelings and hopelessness, these professionals used their knowledge, skills and innovations in the act of caring, which contributed to reconstructing their professional identity.

## RESUMO

**Objetivos:**

analisar o trabalho das enfermeiras retratado na mídia jornalística e seu impacto na construção da identidade profissional da enfermagem.

**Métodos:**

pesquisa qualitativa, retrospectiva, descritiva e documental, com 51 reportagens da Folha de São Paulo. Recorte temporal de março a dezembro de 2020. Análise de Conteúdo Temática foi realizada sob a perspectiva teórica de Claude Dubar. Organização e codificação dos dados foram realizados com o auxílio do *software* ATLAS.ti^®^.

**Resultados:**

emergiram três categorias: *Condições de trabalho na pandemia - um problema que se agravou; Impactos da pandemia no cotidiano de trabalho; Sentimentos gerados pela pandemia*.

**Conclusões:**

apesar de adversidades, como precariedade das instituições de saúde, condições inadequadas de trabalho das enfermeiras, falta de itens básicos de proteção individual, dos sentimentos negativos e desesperança, essas profissionais utilizaram seus conhecimentos, habilidades e inovações no ato de cuidar, o que contribuiu para a reconstrução de sua identidade profissional.

## RESUMEN

**Objetivos:**

analizar el trabajo de los enfermeros retratados en los medios periodísticos y su impacto en la construcción de la identidad profesional de enfermería.

**Métodos:**

investigación cualitativa, retrospectiva, descriptiva y documental, con 51 informes de Folha de São Paulo. Periodo de tiempo de marzo a diciembre de 2020. El Análisis de Contenido Temático se realizó desde la perspectiva teórica de Claude Dubar. La organización y codificación de los datos se realizó con la ayuda del software ATLAS.ti^®^.

**Resultados:**

surgieron tres categorías: Condiciones de trabajo en la pandemia - un problema que se agravó; Impactos de la pandemia en el trabajo diario; Sentimientos generados por la pandemia.

**Conclusiones:**

a pesar de las adversidades, como la precariedad de las instituciones de salud, condiciones de trabajo inadecuadas para las enfermeras, falta de elementos básicos de protección individual, sentimientos negativos y desesperanza, estos profesionales utilizaron sus conocimientos, habilidades e innovaciones en el acto de cuidar, lo que contribuyó a la reconstrucción de su identidad profesional.

## INTRODUCTION

The World Health Organization (WHO) declared COVID-19 as a pandemic on March 11, 2020. Fear with this completely new, complex scenario, with no previous references to the healing and caring process, gripped health workers, in special nursing professionals. The concern with this professional category is due to the fact that, even before the crisis in health services due to COVID-19 materialized, nursing was already experiencing the effects of work precariousness and devaluation^(1)^.

The daily life of nursing is marked by many structural, organizational and working conditions problems, which translate into a lack of work tools, extensive working hours, low wages and poor management of health services. This situation entails numerous consequences for professionals, from physical and psychological distress, absenteeism, illness and even suicide^(2)^. Suffering situations in nurses’ work can be found in other countries, as is the case of a study carried out in Andalusia (Spain), which points out that nursing professionals face a variety of stressful situations on a daily basis, and, to alleviate this stress and suffering at work, a predictive model is developed^(3)^.

The disease’s progress in Brazil was rapid and disastrous, and the Unified Health System (SUS - *Sistema Único de Saúde*) collapsed in several states. With the start of vaccination, the lack of national coordination of the campaign in general caused each state to establish its own rhythm. In addition to restrictive measures being increasingly relativized, it contributed to the virus circulation taking on gigantic proportions^(4)^.

Coping with this pandemic made the historical demands of nursing even more evident regarding professional development, working conditions, working hours, among others^(5)^. Additionally, being on the front line of patient care, the concern for the high risk of contamination increased professionals’ psychological distress. Nurses’ work is crucial and extremely relevant in this pandemic scenario, whether in disease prevention, treatment or recovery of patients, acting in research and, even as volunteers in studies on vaccines^(6-7)^.

The pandemic has rekindled interest and raised many questions regarding nurses’ professional identity, reinforced by the media that highlighted health professionals’ work in all its formats. Communication, as a way of democratizing information, opened an important space for debate and reconsideration about the work of those working on the front lines of the pandemic, and reports in major newspapers and news portals ensured high visibility^(8)^.

The almost daily exposure of nursing work in the journalistic media highlighted nursing professionals’ work, showing the risks and changes in work routine and the important role for nursing and professionals’ evolution and nurses’ social recognition^(8)^. Nursing professionals’ exposure in all media also contributed to a change in society’s view of this profession^(9)^. Nods of appreciation, understanding of what nursing represents in a health institution, also served as support for strengthening nurses’ professional identity.

In this way, we understand that nurses’ professional identity can undergo a reconstruction, given that it is built from characteristics that are shaped through daily work’s historical context and social reflexes, as has been pointed out in several nursing studies^(10-11)^. It is not permanent and can change over time and with everyday contingencies, as is the case with COVID-19. In this regard, professional identity is (re)constructed from interactions in the work environment and successive socializations, being a continuous process^(12-13)^.

Identity is “the result at the same time stable and provisional, individual and collective, subjective and objective, biographical and structural, of the different processes of socialization that, together, build individuals and define institutions”^(12)^. For this reason, we understand that this framework is appropriate to discuss nurses’ work in the pandemic and the way they were portrayed in the journalistic media.

## OBJECTIVES

To analyze the work of nurses portrayed in the journalistic media and its impact on the construction of professional nursing identity.

## METHOD

### Ethical aspects

This study had no direct involvement with human subjects, and the reports are considered in the public domain. Therefore, the need for appreciation by the Research Ethics Committee was waived. This justification is supported under the terms of Resolution 510 of April 7, 2016 and in accordance with Law 12.527/2011.

### Study design

This is documentary research with a qualitative, retrospective and descriptive approach, which used Claude Dubar’s theoretical framework on identity to reflect on the data found. To guide the research, the Standards for Reporting Qualitative Research (SRQR) instrument was used. The SRQR aims to improve the transparency of all aspects of research by providing clear standards for reporting qualitative research^(14)^.

### Study setting

The study scenario chosen to analyze nurses’ work and their impact on the construction of nursing professional identity was the journalistic media, considering the relevance of this vehicle in the context of a pandemic and social isolation to which humanity has been subjected.

### Data source

The primary documentary source used in this study was the newspaper *Folha de São Paulo*, available online, which is considered one of the main means of journalistic communication in the country. The time frame used was the period from March to December 2020, justified by Decree 6 of March 20, 2020 lasting until December 31, 2020, which declared the state of public calamity in Brazil.

The documentary research in this study is based on the feasibility that documents provide to reach objective information within a subjective context of the studied phenomenon’s history. Therefore, it is necessary to systematically use it to explore and process data based on a historical analysis^(15)^.

### Data collection and organization

Data collection in *Folha de São Paulo* was carried out from December 2020 to February 2021, with nursing, covid, pandemic as search terms. The search was carried out using a documental research instrument in journalistic media, crossing the three search terms. We included reports that were related to nurses, that addressed COVID-19 and complied with the objective of this study. We excluded reports that, despite the term “nursing”, were unrelated to the other search terms, i.e., they were job offers, lawsuits, among others. Table 1 shows the result of the search and selection of reports that make up this study.

**Table 1:** Search results and selection of stories in *Folha de São Paulo*, Florianópolis, Santa Catarina, Brazil, 2021

Term	Search terms	N. reports	After reading (filter)
*Enfermeira* (Nurse)	*Covid/pandemia/coronavírus* (Covid/pandemic/coronavírus)	7,755	45
*Enfermeiro* (Nurse)	*Covid/pandemia/coronavírus* (Covid/pandemic/coronavírus)	160	9
*Enfermagem* (Nursing)	*Covid/pandemia/coronavírus* (Covid/pandemic/coronavírus)	436	14
Total		8,351	68

Initially, 8,351 reports were selected, which were read one by one, and after an exhaustive search, 68 reports were selected that met the inclusion criteria. Even so, the reports were read again and 17 other reports were excluded, 12 of which were duplicated, three that contained subjects outside the interest of this investigation and two because they were fake news disseminated by nurses and identified by comparison with the correct ones.

### Data analysis

The study’s final corpus consisted of 51 reports. Data organization and coding were supported by ATLAS.ti^®^ 9.5.0, according to the following steps: 1. Pre-analysis - exhaustive reading of information and initial coding of citations, where simple codes were created that identified the reports’ general context (e.g., lack of Personal Protective Equipment (PPE), distance from family members, fear of contamination); 2. Material exploration - search for determining aspects or words and structuring into analysis categories/code groups, in which the primary codes were associated with analysis categories (e.g., lack of PPE - working conditions); 3. Data treatment and interpretation - final organization of the information obtained and creation of networks, in which categories were divided and made it possible to create networks of meaning^(16)^. At this time, the Thematic Content Analysis proposed by Bardin^(16)^ was carried out, analyzed from the perspective of Claude Dubar theoretical framework (2005)^(12)^.

The content of analyzed contents was kept in full, but without identifying people; therefore, the extracted contents are identified by the letter R for the report followed by the ordinal number assigned by ATLAS.ti^®^ when inserting the reports in the software. We emphasize that the analysis of identity during the COVID-19 pandemic is closely linked to the national scenario. Despite this, some reports published in *Folha de São Paulo* refer to the international context and, as they comply with the theme, they were included.

## RESULTS

The results were divided into three categories, considering the reported content and their relationship with aspects that influence professional identity during the COVID-19 pandemic. They are: *Working conditions in the pandemic - a problem that has worsened; Impacts of the pandemic on daily work; Feelings generated by the pandemic*.

### Working conditions in the pandemic - a problem that has worsened

**Figure 1:**
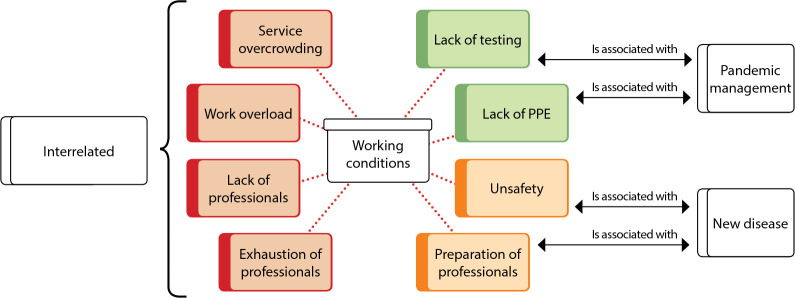
Working conditions revealed in the COVID-19 pandemic, Florianópolis, Santa Cataria, Brazil, 2021

*Source: network of meanings extracted from ATLAS.ti^®^.*

Nursing working conditions have always been a problem, however, with the pandemic, they have become even more evident, with emphasis on problems related to health service management, as shown in Figure 1.

The analyzed content shows that nurses focus their speeches on the working conditions offered, which, since the beginning of the pandemic, have been marked by the overcrowding of health services across the country. The unpreparedness to face this new situation brought consequences for health professionals in general and for nursing professionals in particular for numerous reasons that can be seen in the reports as follows:

*So many people arrived that, when the serum was going to be put in, patients practically died at the hands of professionals, who started to discharge or send patients to other health centers to free up beds.* (R20)

*The hardest moment was at the beginning of the pandemic. It felt like I was in a horror movie due to the amount of people arriving at the hospital at the same time.* (R26)

[...] *the pandemic highlighted problems that preceded the health crisis, such as the lack of interest of hospitals in hiring more professionals in the area, the lack of public tenders and poor working conditions in the peripheries and in the countryside of Brazil.* (R43)

*It’s always that terror shift. One hour, one has to intubate, the next, another has to defibrillate. We don’t stop.* (R24)

*Under normal conditions, each nurse takes care of six patients, but there are days when we see 12. As a geriatric hospital, we take care of many older adults who need assistance at all times, someone by their side to eat, drink water and any other need activity.* (R53)

*The psychological issue has caused a great impact. The physical and mental overload has been absurd. People report burnout.* (R25)

With regard to problems aggravated by the pandemic management, the lack of PPE and efficient testing of the population made nurses lead moments of outbursts in front of the media; more than that, claims have become threats to stop services.

*Professionals working at the hospital told the report that there are not enough surgical or N95-type masks, nor enough aprons, gloves and hats for employees. Today a patient arrived with all the suspected coronavirus and we simply have few common masks. A co-worker, with a child and an older adult at home, asked the supervisor for the N95 and was denied. The other co-worker insisted, because she belongs to the risk group, she has asthma. The mask was thrown at her, as if it were a favor.* (R5)

*Nurses working in health units in Pernambuco threatened to go on strike. They denounce the lack of basic protective equipment, such as masks and aprons, and shortages of alcohol gel and soap in hospitals.* (R7)

*There are reports of professionals who work 12-hour shifts without taking off their protective clothing because they have no other, in case they go out to eat, or of people who are using diapers to avoid having to go to the bathroom.* (R28)

Other countries have also reported problems regarding PPE, such as China and the United States.

*In China, working conditions have reached extreme levels. Nurses in the Wuhan district, epicenter of coronavirus in the country, cut their hair and shaved their heads due to a lack of supplies and protective equipment.* (R6)

*Co-workers of a nurse at the hospital are also angry. Some complained on social media that they didn’t have enough protective clothing or masks.* (R9)

With all these associated problems, nurses report that inadequate working conditions contribute to making them increasingly insecure, especially due to the lack of preparation in the face of a new and highly transmissible disease.

*At the hospital, a nurse received me without much information. It’s all new and you feel everyone’s insecurity.* (R1)

*They say that nurses are prepared for bad experiences, but I don’t agree. At the university, we are not adequately prepared for this, it is very difficult to manage the pain.* (R10)

*The regions that had less training, less government support are the ones where professionals feel less prepared.* (R33)

The set of information from analysis of reports demonstrates a clear relationship between precarious working conditions and poor pandemic management, highlighting, above all, the impact of working conditions on nursing professionals.

### Impacts of the pandemic on daily work

**Figure 2:**
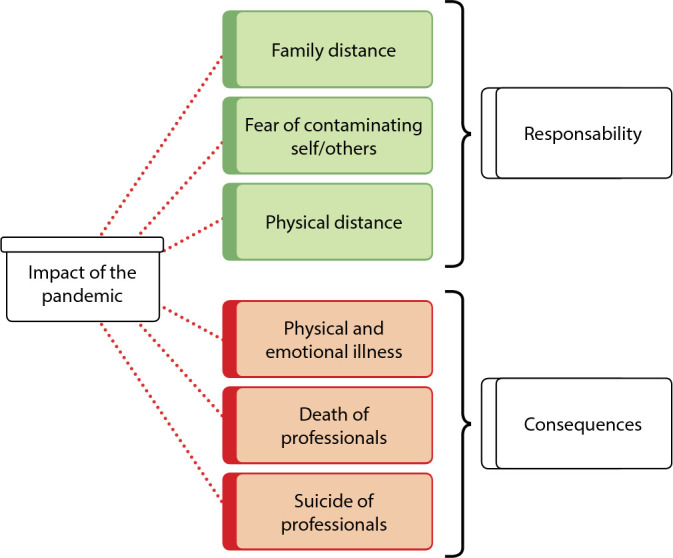
Impact of the COVID-19 pandemic on nurses’ work, Florianópolis, Santa Cataria, Brazil, 2021

*Source: network of meanings extracted from ATLAS.ti^®^.*

This category concerns the impacts that the pandemic brought to work, which, in general, was already very complex, and which takes on another, much more chaotic dimension than imagined. The consequences of the pandemic, related to family illness, physical separation from the family, death of co-workers, deeply affected daily work routine, in which, at any moment, there could be an imminent contamination, or death of a family member, friend or co-worker. What was revealed in the reported content were two dimensions of the impact of the pandemic: one related to nurses’ responsibility and another related to consequences, which can be devastating, as shown in Figure 2.

Among the most significant impacts of the pandemic, nurses reported the issue of physical distancing from patients as well as from family and friends (PPE use made physical contact more difficult). This withdrawal was motivated by the fear of contaminating themselves or of contaminating the people around them, and continuous stress during the daily work process.

*One thing that COVID-19 has changed is the senses. They operate completely differently. You wear topcoats and a cap, which affect your hearing. You hear everything muffled. The masks, very tight on the face, do not allow you to perceive any odor. You feel your own breath, but no smell from outside. Over the eyes, a mask or glasses, and even the view has to adapt. On the hands, always two pairs of gloves, and the touch also disappears. We had to start all over again to learn to use our senses.* (R19)

*Nurse* [nurse’ name], *29 years old, was forced to change her house in Peruíbe* [137 km from São Paulo], *where she lives with her husband, for a hotel room offered by Hospital Sírio-Libanês, in Bela Vista* [Center region], *where he works. In the hotel, there is no interaction with other people. We are still in social isolation. We ate inside the room, she says. To avoid loneliness, the nurse uses the internet. Technology has helped. I speak with my family. People know that I’m going through this situation and support.* (R17)

*It’s the hardest part for me. I’m going to spend Mother’s Day without them* [the children], *but that’s life. I feel like I’m missing out on moments with them, because every day I talk to them it feels like they’re different.* (R27)

The consequences of the pandemic take nurses’ speeches to a very delicate dimension: physical and emotional illness. But some reports dealt exclusively with the death of health professionals, especially nurses, most of them due to the virus, others due to the impact that mental distress brought to the daily professional practice, since they did not have time to treat themselves.

*At least 8,265 health professionals across the country are away from their duties amid the new coronavirus pandemic. These employees had to leave work because they showed symptoms of the disease or because they are part of a risk group.* (R14)

*This is especially necessary in places where co-workers have passed away.* [Institution’s name] *has an entire traumatized team. Of 11 co-workers, two of them died. They feel death very close* [...] *there are still no studies on this, but the perception of entities and specialists is that, with the increase in deaths of co-workers from the disease, combined with the fear of contagion and the exhausting journeys, emotional and psychiatric conditions tend to get worse.* (R25)

[Nurse’s name] *said he had an anxiety attack. I woke up in the middle of the night with severe chest pains. I thought I was having a heart attack. I went to the doctor, but it was an upset stomach caused by my high stress level.* (R48)

*She regrets not being able to comfort families when one of them dies. “Before, we used to give hugs. Now, they can’t even approach us. Even patients’ clothes are discarded”, she says that, during the pandemic, she already had a professional co-worker who died in her arms.* (R23)

*Brazil accounts for 38% of deaths of nurses worldwide. At least 260 of these professionals have died, according to the International Council of Nurses. We have already passed to the USA, which recorded 91 deaths in nursing.* (R28)

Two reports published in *Folha de São Paulo* also pointed to the suicide of nurses in Italy and China.

*Nurse commits suicide in Italy after being diagnosed with coronavirus.* (R8)

*Death of a nurse in China draws attention to a professional category in crisis. The circumstances of the incident remain unclear, but police later said the death of* [nurse’s name], *28, needed to be assessed by “departments specializing in public mental health”. In a country where deaths are rarely declared suicides, this is a strong indication that the nurse, a married mother of one, probably killed herself.* (R44)

The impacts generated by the pandemic are felt by nurses throughout the time they are on the front line in caring for the population that fell ill from the virus. It should be noted that these impacts will still be felt in the medium and long term, due to disease severity and the way the pandemic was managed over time.

### Feelings generated by the pandemic

**Figure 3:**
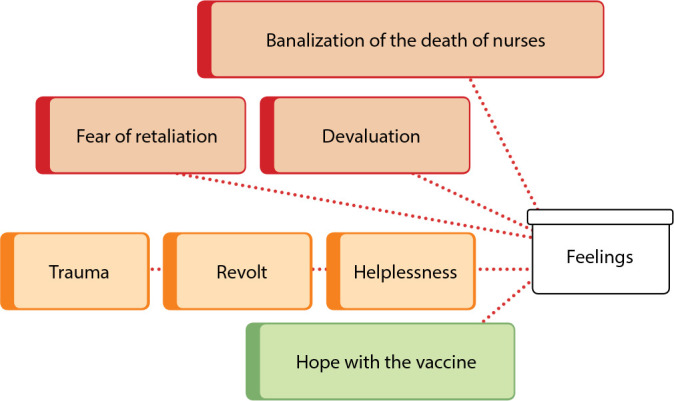
Feelings generated by the COVID-19 pandemic, Florianópolis, Santa Catarina, Brazil, 2021

*Source: network of meanings extracted from ATLAS.ti^®^.*

This category deals with the feelings revealed by nurses, in the face of so many adversities encountered in professionals’ work in relation to the pandemic, as shown in Figure 3. The predominant feelings are of a negative nature and reflect the influence on their professional identity, as they are a portrait of how the society see them. Therefore, helplessness, revolt and trauma reflect the anguish experienced by the disease itself and by the inefficient management of health services. Finally, and not least, something positive is revealed, hoping for better days with the arrival of the COVID-19 vaccine.

Feelings of devaluation and trivialization of the deaths of nurses are closely related to working conditions and the lack of recognition, by society, about the work developed by them in their daily lives.

*Even with the quarantine and preventive measures, hospital officials told Folha that they have been stigmatized and even criticized by part of the population because of the concentration of cases among health professionals.* (R14)

*The issue of low wages even interferes with work quality.* (R31)

[Nurse’s name] *is a nurse, in a country where every minute a health professional is infected, especially nursing technicians and nurses.* (R45)

*More than two months without seeing my parents, more than 23 thousand lives lost, many people still fighting in the ICU, companions who lost their lives, the rest struggling every day with this fear of contaminating us, thousands of people losing their jobs* [...] *I had a slight hope that something would change.* (R21)

*The Wuhan Union released a brief public statement in response to the death of* [a nurse who committed suicide in China], *saying that it was actively cooperating with the family and relevant authorities to deal with the aftermath of this incident. XXX’s family said in an interview that a request to meet with hospital officials to discuss their daughter’s death was turned down. The family continues to struggle to find out the truth of the facts.* (R44)

Fear of retaliation refers to insecurity in portraying negative aspects of work that are poorly managed by health services and, which often, when talking about these topics, can lead to administrative penalties and even dismissal from the position they occupy.

*Two nurses, who requested anonymity to speak for fear of being fired, said they are disposable gowns made of permeable material, which is why the nurses wrapped them in garbage bags. They said the photo was taken on March 17, at a time when there were many coronavirus patients in the hospital and others who had not yet been tested but were showing symptoms of the infection.* (R9)

*The 35-year-old nurse agreed to speak by phone, but declined to be identified for fear of being fired.* (R20)

Faced with so many misfortunes caused by the pandemic, nurses portray in the reported content, the trauma associated with the large number of deaths, the feeling of helplessness due to the constant suffering witnessed and revolt.

*Sometimes, some of us break down: we feel despair and we cry because we feel powerless when our patients are not getting better.* (R3)

*Every day that I see the streets more crowded with people, I think there will be more days away from my daughter.* (R13)

*Hard to see someone dying without being able to do anything. We are prepared to save lives, not to watch mortality as high as in an epidemic.* (R17)

*His death could have been avoided, I’m disgusted. He was healthy* [on the death of a fellow nurse infected with the virus]. (R9)

*I was dominated by a feeling of disillusionment, of abandonment. How is it possible to fight to take care of people if part of the population attacks us?* (R28)

However, as a way of establishing a perspective for the future, a light at the end of this tunnel, the vaccine comes as an encouragement, a hope for better working days, in which nurses can see their actions socially recognized and valued.

*Now I also have hope because I want everyone to be vaccinated.* (R51)

*The first professional from the municipal health network to receive the vaccine against COVID-19 describes the new phase with one word: relief.* (R45)

*I hope everyone is vaccinated quickly, we need to achieve herd immunity and control this pandemic soon.* (R30)

Although negative feelings prevailed, there is still hope in nurses’ discourse, and this is directly related to the vaccine.

## DISCUSSION

The portrayal pointed out by the journalistic media regarding nurses’ work during the COVID-19 pandemic showed issues far beyond the daily work experienced daily by these professionals. What was evident in the reports analyzed during this period was the impact of working conditions and the feelings experienced during the pandemic, due to the total lack of knowledge about how to deal with coronavirus. Health professionals’ work, and in particular nurses’, made visible by society as a whole and even in hospital institutions, allowed analyzing the impact of the pandemic in terms of the construction of a new professional nursing identity, related to the present moment and nurses’ empowerment in decision-making in their professional work environments.

Nurses’ work, especially in hospital units, remained invisible and undervalued, generating suffering and frustration. This invisibility of care may be linked to the numerous activities they develop in hospital dynamics and to a certain limitation of their field of action, associated with low pay, work overload and the lack of incentives for training^(17)^. This contributes to a fragile identity exposed to the type of work nurses are developing.

This statement is in line with Dubar’s conceptions^(12)^, that the whole process of identity formation (construction, deconstruction and reconstruction) goes through the relationships in the environment in which people are inserted. The formation of identity is based on the result of successive socializations. In this regard, the pandemic has profoundly marked the nursing profession, especially due to the impacts of the successive narratives and contexts present in nurses’ work, in a mix of experiences, good, bad and overcoming the challenges faced.

Precarious working conditions were already present in all nursing services, especially in hospitals, and come from different and complex factors, such as shortage of professionals and material resources, work overload, strenuous working hours, fragile work relationships and wages far below the target^(2,18-19)^. Nursing professionals are faced with many occupational diseases, with different workloads acting on each other at the same time, which can cause different damages to health^(20)^.

The work environment in which nursing is inserted during the pandemic can bring with it many risk factors, either because of the number of complex procedures or because of the environment itself, which often resembles a confinement very similar to that experienced on oil platforms^(21)^.

Historical problems regarding nursing working conditions gained even more worrying proportions with the pandemic, which, given the overcrowding of services and the lack of adequate dimensioning, led professionals to exhaustion, compromising care especially in highly complex sectors^(22)^. Lack of nurses and the increase in the degree of complexity during the pandemic, together with the high rates of absenteeism and turnover, made the reality of services more difficult^(22-23)^. This issue had already been raised and highlighted by the profession’s control bodies. This reality directly reflects on patient safety issues, as they influence unfavorable outcomes, increasing the incidence of adverse events^(22-23)^.

Still on working conditions during the pandemic, vehemently reiterated in the analyzed reports, lack of PPE and testing for COVID-19 are determining factors in nurses’ speeches, showing indignation regarding the central management of the pandemic. All processes for acquiring materials and equipment at the time of the crisis were slow and generated a lot of anger by health professionals, especially nursing and medicine, already stressed with work overload and the dangers of contamination for themselves and their families. The decisive role for the pandemic to become, practically, a catastrophe, came, in part, from the Federal Government^(24)^ as well as the failure to comply with existing recommendations and public health policies that could help in conducting it.

As hospitals reached their maximum capacity, many essential supplies such as drugs, beds, PPE became scarce. In addition to this, an aspect reported in studies that had a negative effect on nurses’ physical and emotional health was the scarcity and discomfort of prolonged use of PPE in the ICU, affecting quality of work^(25-26)^. One study even reported the need for nurses themselves to buy the PPE necessary for care^(27)^.

Brazil’s dependence on the foreign market for the acquisition of equipment and the predatory behavior of other countries, caused the biological risk to increase significantly among nurses^(22,28)^. Lack of PPE and lack of trained health professionals to care for the large number of COVID-19 patients are risk factors that compromise nurses’ and patients’ safety and well-being^(29)^.

Even in the face of this scenario, nurses played a leading role in the pandemic, in all contexts, from Primary Health Care to the most complex hospital, assuming the risks of contamination along with precarious working conditions^(30)^. This role does not go unnoticed for the construction of a new professional identity.

Considering everything that nurses did during the crisis period, it is necessary to identify the construction of a new professional identity, given that nurses have a new conception of themselves and the world, as Dubar points out^(12)^.

It is important to highlight that, given the capacity for hospital care around the world, including Brazil, and the high rates of transmission and illness in the population, the probabilities of saturation were estimated with some precision and in the short term. In Brazil, studies were aimed at estimating the health system’s collapse, by macro-region and in Brazilian capitals, taking into account the number of beds, respirators and health professionals^(28,30-31)^.

Several studies show that there were many health professionals who fell ill with COVID-19, and in countries such as China, Spain and Italy, 13 to 20% of those infected were health professionals, with a high number of deaths among nurses^(32-34)^. In Brazil, the reality is not very different. Official data from the Federal Nursing Council, through the Nursing Observatory, reveal that, until October 4, 2021, 58,725 cases were reported, with 866 deaths being nursing professionals^(35)^.

This problem may have been aggravated by the way the pandemic was treated, from the beginning, by the Federal Government and the Ministry of Health. By defending a denialist policy and underestimating coronavirus’ potential, many behaviors were taken late, or even neglected^(36-38)^. The lack of efficient management focused on people made the pandemic gain strength in the country, being a “nest” of variants, increasingly transmissible, and that brought catastrophic results for the country, which already accumulates 30,210.85 million cases and 661,656 thousand deaths^(39)^.

Given this context, nurses began to reinvent themselves, reviewing protocols for wearing PPE, protocols for the care of critically ill patients, including within educational institutions, innovating in care technologies, involvement in research and mass participation in vaccination campaigns^(40)^. Moreover, they used creativity and social media resources to help educate society, asking people to stay at home and teaching them how to properly wash their hands^(8)^. Moreover, the role of nurses as advocates for patients (advocacy) is highlighted, seeking to ensure their safety, respect for values and providing comfort in the final moments of the lives of the people under their care^(41-42)^.

The fear of contamination of professionals, patients and family members made many nurses move away from living with their families and feel distant from patients. The pandemic revealed that nurses, despite feeling unprepared and insecure in relation to the new disease, highlighting this issue in analyzed reports, they did not give up, did not abandon their posts and faced the problem head on. Thus, a new type of pride is configured, of professional and collective self-perception, which can indeed reaffirm the individual and collective professional identity. However, fear was replaced by the search for new strategies for care, being a new professional identity, seen through the use of new technologies and innovating care processes (telemarketing, telemonitoring, identification methods superimposed on PPE, among others), with the intention of shortening distances and continuing to guarantee the safety of care for oneself and others^(41)^.

Faced with so many adversities provoked or even evidenced by the pandemic, especially before the possibility of prevention provided by vaccines, many negative feelings were expressed by nurses in the analyzed reports. These feelings, such as trauma and revolt, were motivated by deaths of co-workers, devaluation of profession and feeling of helplessness generated by these circumstances. This feeling of being more vulnerable, together with the fear of being contaminated, has a dramatic impact on nurses’ psychic and cognitive functioning^(43-44)^. Nursing devaluation as a health profession, as important as the others, is not a new topic and did not emerge with the pandemic.

With the passage of time and analysis of reports, negative feelings and a sense of hopelessness gave way to confidence that the pandemic is losing strength, especially with the arrival of vaccines and consequent reduction in the number of deaths and contaminated. Faced with a new scenario, nurses are more empowered, with a sense of accomplishment, but without losing sight of the reconstruction of their identities^(45)^, who left the popular imagination of angels and heroines and moved to an identity that denotes the importance of science and the appreciation of those who are always with the population in humanity’s worst moments.

“Identity is never given, it is always constructed and will have to be (re)constructed in greater or lesser and more or less lasting uncertainty”^(12)^.

The fight for recognizing the importance of nursing for the care and appreciation of the category is an old movement, which corresponds to the social image that the institution builds through representations of what it is to be a nurse. In this regard, it is clear that the pandemic has positively impacted a new professional identity for nurses, not only because of the actions already reported, but also because of the visibility of their work in society and their importance in caring for and fighting the disease.

Nurses’ and nursing team’s work is fundamental to guarantee the health and well-being of all, due to scientific knowledge and diversity of contexts in which they work. However, nurses’ work is not disconnected from the numerous adversities found in the health sector. In the face of the pandemic, their social importance for health prevention, promotion, treatment and recovery was emphasized.

### Study limitations

We understand that one aspect that may have limited this study was the use of a single document source, despite the fact that *Folha de São Paulo* is considered one of the most important media vehicles in the country. A complicating aspect of the journalistic search system is that it does not allow using AND/OR Boolean search engines, limiting the search for keywords individually.

### Contributions to nursing, health, or public policies

This study brings relevant contributions to nursing considering that it presents a broad discussion about nurses’ work during the pandemic, the way they perceived themselves during the journalistic interviews, allowing a reflection on how we want society to perceive the nursing profession. The study also presents subsidies for new studies related to professional identity during and after the pandemic as well as future research and reflections on nursing education. This study also records the struggle of a class, contributing to the preservation of the profession’ memory and the pandemic history.

## FINAL CONSIDERATIONS

The results of this study demonstrate the impact of the pandemic on the construction of nurses’ professional identity, from coping with the need to face the installed conflict, with propriety and remedy the situation using their knowledge, skills and implementing new technologies and innovations in the act of caring.

The empowerment of nurses, reinforced by fighting against the adversities of the pandemic, generated visibility in society, strengthened health professionals and, in particular, nursing, contributing to a new professional identity being evidenced, based on the value of and for the work performed.

Regarding using journalistic media in this study, it is understood that this can and should be an effective source to identify different realities of daily work and give visibility to the profession, largely forgotten and invisible before the pandemic. Nurses also learned to use this communication mechanism, to be present and to fight for their rights.

The pandemic pointed to the urgent need to make visible not only nurses, but all Brazilian nursing, in order to ensure more social recognition and simple tributes, in addition to public policies aimed at improving working conditions, as well as workers’ quality of life, and that if these are perpetuated.

It is inevitable to recognize the proportions of social recognition for nursing professionals. The media space leveraged by the pandemic reached a significant portion of the population, which started to have new perspectives and understandings about roles. Such recognition had a motivating effect for workers who, amid so many confrontations, felt socially recognized for their commitment to care with technical-scientific knowledge and human skills.
